# Comparative analysis of metacyclogenesis and infection curves in different discrete typing units of *Trypanosoma cruzi*

**DOI:** 10.1007/s00436-024-08183-4

**Published:** 2024-04-11

**Authors:** Tatiana M. Cáceres, Lissa Cruz-Saavedra, Luz Helena Patiño, Juan David Ramírez

**Affiliations:** 1 Centro de Investigaciones en Microbiología y Biotecnología-UR (CIMBIUR), Facultad de Ciencias Naturales, Universidad del Rosario, Bogotá, Colombia; 2 Molecular Microbiology Laboratory, Department of Pathology, Molecular and Cell-Based Medicine, Icahn School of Medicine at Mount Sinai, New York, NY 10029 USA

**Keywords:** Chagas disease, *Trypanosoma cruzi*, Discrete typing units, Genetic diversity, Metacyclogenesis

## Abstract

Chagas disease (CD), caused by the complex life cycle parasite *Trypanosoma cruzi*, is a global health concern and impacts millions globally. *T. cruzi*’s genetic variability is categorized into discrete typing units (DTUs). Despite their widespread presence in the Americas, a comprehensive understanding of their impact on CD is lacking. This study aims to analyze life cycle traits across life cycle stages, unraveling DTU dynamics. Metacyclogenesis curves were generated, inducing nutritional stress in epimastigotes of five DTUs (TcI (MG), TcI (DA), TcII(Y), TcIII, TcIV, and TcVI), resulting in metacyclic trypomastigotes. Infection dynamics in Vero cells from various DTUs were evaluated, exploring factors like amastigotes per cell, cell-derived trypomastigotes, and infection percentage. Statistical analyses, including ANOVA tests, identified significant differences. Varying onset times for metacyclogenesis converged on the 7th day. TcI (MG) exhibited the highest metacyclogenesis potential. TcI (DA) stood out, infecting 80% of cells within 24 h. TcI demonstrated the highest potential in both metacyclogenesis and infection among the strains assessed. Intra-DTU diversity was evident among TcI strains, contributing to a comprehensive understanding of *Trypanosoma cruzi* dynamics and genetic diversity.

**Supplementary Information:**

The online version contains supplementary material available at 10.1007/s00436-024-08183-4.

## Introduction

Chagas disease (CD) poses a substantial public health concern, affecting an estimated 6 to 7 million people globally, with approximately 71 million at risk of contracting it. The causative agent of CD is the protozoan parasite *Trypanosoma cruzi* (Rassi et al. 2010; World Health Organization = Organisation mondiale de la Santé, 2015).

*Trypanosoma cruzi* displays extensive intra-specific genetic diversity, classified into six subgroups known as discrete typing units (DTUs) designated as TcI-TcVI, with the potential addition of a seventh DTU, TcBat, associated with bats but documented in human infection cases (Ramírez et al. 2014; Zingales et al. 2009, 2012). These DTUs are widely distributed throughout the Americas in various ecotopes (domestic, peridomestic, and sylvatic) and can coexist in the same vector and host (Brenière et al. 2016; Velásquez-Ortiz et al. 2022). While there has been extensive research in the molecular epidemiology field of *T. cruzi*, studies exploring the impact of genetic diversity on transmission cycles, geographic distribution, and clinical manifestations of CD are scarce, and conclusive associations have not been firmly established (Jiménez et al. 2019; Messenger et al. 2015; Zingales 2018; Zingales et al. 2009). Nevertheless, the exploration of genetic diversity has provided valuable insights into the intricate interplay between host and pathogen and the genetic factors that influence the evolution of the disease (Zingales & Macedo 2023).

Marked genomic variability and aneuploidy have been observed among different DTUs, even within isolates of the same DTU. This variability may play a crucial role in adaptive environmental responses. Then, it is essential to understand how these genetic differences manifest in specific phenotypic characteristics, such as virulence, replication rates, tissue tropisms, metacyclogenesis potential, parasitemia curves in animal models, and treatment resistance (Berná et al. 2018; Cortez et al. 2022).

Among the mentioned characteristics, metacyclogenesis holds particular importance—an essential and critical process for *T. cruzi* transmission. During this stage, the non-infectious stage known as epimastigotes transforms into an infectious stage, called metacyclic trypomastigotes (MTs) in the rectal ampulla of the vector insect. These MTs acquire the capability to infect mammals once released in the insect vector’s feces (Gonçalves et al. 2018). Despite identifying specific genes associated with this stage, significant morphological variations have been observed among different DTUs (Abegg et al. 2017; Avila et al. 2003). Thus, suggesting that the genetic diversity of the parasite may play a crucial role in its ability to evolve and spread in the natural environment, thereby influencing transmission rates.

In addition to identifying changes in metacyclogenesis, it is crucial to describe differences in the infection dynamics of *T. cruzi* in the host, especially concerning genetic diversity. Variations in infectivity, motility patterns, recognition efficiency, and cell invasion have been observed. Although the precise mechanisms underlying these differences remain uncertain, the possible involvement of virulence factors such as trans-sialidases, mucins, mucin-associated surface proteins, and exovesicles is postulated (Arias-del-Angel et al. 2020; Medina et al. 2018). The expression of the aforementioned proteins is heterogeneous in terms of diversity and copy number in different *T. cruzi* genotypes, potentially impacting the virulence and pathogenicity of the genotypes (Burgos et al. 2013).

The above observations suggest variations in the behavior of cell-derived trypomastigotes (CDT) during infection, indicating that the infectious performance may differ not only among the parasite’s DTUs but also among specific strains (Arias-del-Angel et al. 2020; Zingales 2018). The potential link between the parasite’s genetic diversity and critical aspects such as CD epidemiology, DTU geographical distribution, clinical manifestations, and phenotypic characteristics like treatment resistance, host diversity, and the involvement of vectors in disease propagation emphasizes the need for research providing a more comprehensive understanding of this complex relationship.

Therefore, the primary aim of this study was to evaluate variations in metacyclogenesis potential and in vitro infection differences among different DTUs of *T. cruzi*: TcI (MG), TcI (DA), TcII(Y), TcIII, TcIV, and TcVI*.* This was achieved through the development of metacyclogenesis and infection curves, followed by statistical comparisons.

## Materials and methods

### Parasite cultivation

Epimastigotes of MHOM/CO/01/DA (TcI), MHOM/CO/04/MG (TcI), MHOM/BR/53/Y (TcII), MT3663-845 (TcIII), JJ-85 (TcIV), and TULAHUEN CL98 (TcVI) were cultivated for the metacyclogenesis curve. Genotype confirmation was achieved through Sanger sequencing of the 18 s and mini exon genes, aligning them with reference genomes. Numerous attempts were made to infect Vero cells with TcIII DTU; however, none yielded the required CDT quantity to attain the standardized infection radius specified in this study. Consequently, the assessment of infection dynamics for this DTU was not feasible. Moreover, experiments with DTU TcV were not undertaken due to the unavailability of this strain at both our research center and our collaborators’ facilities. The metacyclogenesis curve was developed in LIT medium with 10% fetal bovine serum (FBS) and 1% penicillin/streptomycin, incubated at 26 °C with 1 × 10^8^ parasites/mL in the logarithmic phase. Three biological replicates were performed per DTU for 12 days, following the standardized temporal interval by Cruz-Saavedra et al. (Cruz-Saavedra et al. 2017).

### Parasite count and identification of MTs

The parasite concentration was monitored over 12 days using the Neubauer chamber counting method. For the metacyclic trypomastigote (MT) identification and quantification, 100 parasites were counted using Field’s staining, with three technical replicates for each biological replicate. These forms were distinguished based on the position of their kinetoplast, nucleus, and flagellar modifications. Epimastigotes present a compact nucleus in the middle of the cytoplasm, a kinetoplast located in the anterior part of the parasite, followed by the flagellum. On the other hand, metacyclic trypomastigotes show an elongated nucleus, a kinetoplast located in the posterior part of the parasite, and a flagellum that surrounds the cytoplasm of the parasite from the posterior to the anterior section. From these data, a percentage was calculated and applied to the total parasite count per day, allowing for the daily quantity of MTs produced. This methodological approach was previously employed by Abegg and colleagues (Abegg et al. 2017; Cruz-Saavedra et al. 2017).

### Purification of MTs

We followed the protocol standardized by Cruz-Saavedra et al. (2017), to purify MTs. The procedure involved ion-exchange chromatography on a sepharose-DEAE, where epimastigotes cultivated for 10 to 12 days passed through the system, resulting in MTs present in the eluate. Finally, the obtained forms were observed using Field’s staining (Cruz-Saavedra et al. 2017).

### Generation of Cell-Derived Trypomastigote from Cells and Infection

Vero cells (Vero, ATCC® CCL-81™) were cultured in RPMI medium supplemented with 10% FBS and 1% antibiotic–antimycotic. Cells were incubated at 36 °C in a 5% CO_2_ atmosphere for 24 h. Purified MTs were cultured with semi-confluent Vero cells at a ratio of five parasites per cell in a cell culture flask of 25 cm^2^. The incubation continued under the same conditions for 12 days, with daily microscopic evaluations. Upon the evident production of CDTs, these were utilized for subsequent infections.

### Infection Assessment

For the infection curve construction, two study groups were established: a control group with a concentration of 30,000 Vero cells/mL in 24-well plates and an infected group with the same concentration of cells exposed to 15 CDTs of *Trypanosoma cruzi* per cell. After 24 h of exposure, the supernatant was washed with PBS to remove parasites, and a fresh medium was added. Both groups had three replicates and were evaluated at five time intervals: 24, 48, 72, 96, and 120 h (Oliveira et al. 2017).

The infection was conducted using TcI (MG), TcI (DA), TcII, TcIV, and TcVI at intervals of 24, 48, 72, 96, and 120 h. In the initial phase, the supernatant from each well was removed, and CDTs were counted using a Neubauer chamber. Subsequently, cells were washed with PBS and fixed with 8% formaldehyde, followed by incubation at room temperature and Field’s staining. The plates were observed under an inverted microscope, photographs were taken at 40 × , and analyses were performed using ImageJ software with the “Sharpen” function to count amastigotes per cell in 300 cells.

### Statistical analysis

The software “Prism-GraphPad” was employed to generate a comparative graph illustrating the metacyclogenesis and infection curves of all analyzed DTUs. Data normality was assessed using a Shapiro test, followed by a one-way ANOVA at a significance level of 0.05 to determine the initiation days of metacyclogenesis. Furthermore, a two-way ANOVA was conducted to evaluate differences between DTUs on the analyzed days.

## Results and discussion

**Fig. 1:**
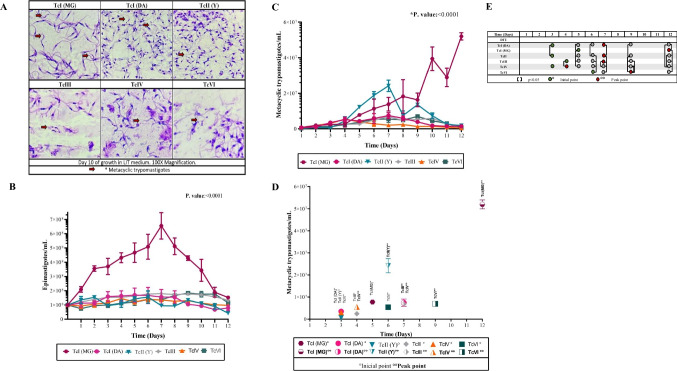
**A** Microphotographs of 10-day incubation cultures of *Trypanosoma cruzi* epimastigotes in LIT medium with Field’s staining and magnification (100 ×), metacyclic forms are indicated by red arrows. **B** Epimastigote production, evaluated for 12 days in a LIT medium. **C** Metacyclogenetic curve representing the dynamic process of metacyclic trypomastigote formation in *T. cruzi*. In *x* is the time (days) while the *y*-axis indicates the number of metacyclic trypomastigotes (MT) per milliliter. The data points provide information on the efficacy and kinetics of metacyclic trypomastigote production. **D** Dynamics of metacyclogenesis in *T. cruzi*, capturing both the starting point and the peak of the process. The data presented provide a better understanding of the timing and efficiency of metacyclic trypomastigote production. **E** This comparative approach uses brackets to indicate statistically significant differences (*p* < 0.05) between DTUs in both the onset and peak phases of metacyclogenesis. In this graph, it can be seen whether there are DTUs with noticeable variations during the kinetics of metacyclogenesis

The induction of metacyclogenesis is triggered by nutritional stress, activating the differentiation of epimastigotes into infective forms of the parasite (Avendaño et al. 2006). The differentiation process encompasses notable morphological changes, with observed variations during the metacyclogenesis process in DTUs (Figueiredo et al. 2000; Gonçalves et al. 2018). Although variations were noted in this study (Fig. 1A), further microscopic analyses using complementary approaches are essential to discern the significance of these variations and explore potential ultrastructural changes (Abegg et al. 2017; Gonçalves et al. 2018). Throughout the construction of the growth curves of epimastigotes, a consistent trend was noted among the various DTUs, differing from the multiplication curve for TcI (MG), where a higher production was observed within the same timeframe (Fig. 1B).

**Fig. 2:**
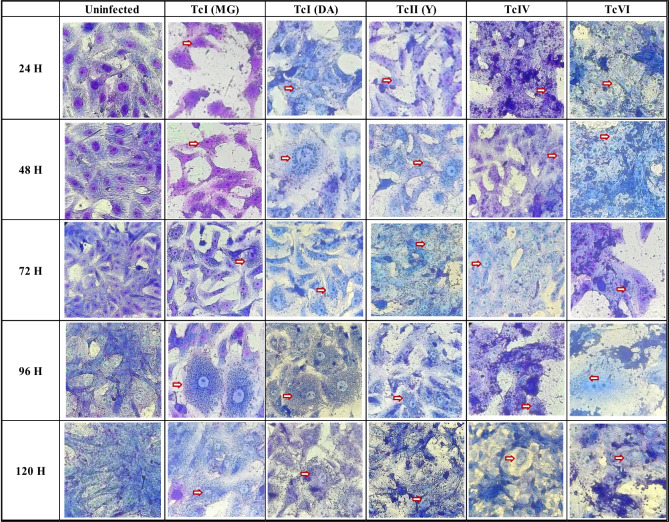
Mosaic of microphotographs showing *T. cruzi* CDT infections in Vero cells for 120 h, using different DTUs. Samples were fixed with formaldehyde and stained with Field’s stain. This representation captures the different stages and dynamics of infection in the different DTUs, and the cell morphological changes induced. White arrows indicate the presence of amastigotes at 24 h. By 72 h, amastigote nests become visible, and at 96–120 h, nests along with cell-derived trypomastigotes are observed

**Fig. 3:**
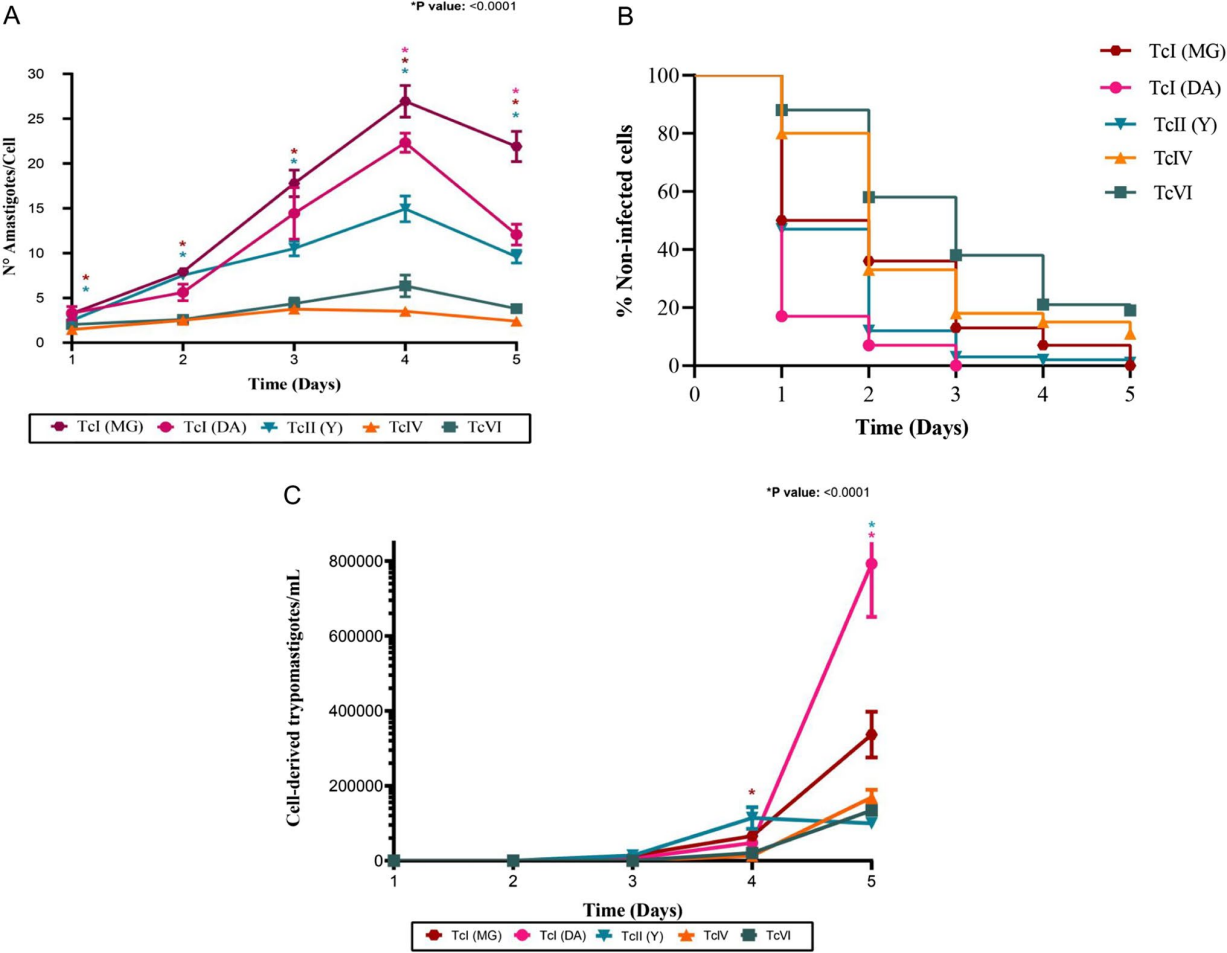
**A** The production of amastigotes per cell in *T. cruzi* infections provides insight into the intracellular dynamics of *T. cruzi* within host cells. **B** Percentage of uninfected cells observed along the infection curves, susceptibility dynamics, and infection progression, highlighting variations in the percentage of uninfected cells throughout the experimental period. **C** Release of cell-derived trypomastigotes during infection of Vero cells, Neubauer chamber count

The onset of metacyclogenesis occurred between the 3rd and 6th day, lasting approximately 6 to 7 days. Similar levels of MTs were observed at peak points for TcI (DA), TcIII, TcIV, and TcVI (Fig. 1C, D; Table S1). TcIV exhibited the lowest production of metacyclic forms (Fig. 1D, E) and demonstrated low efficacy in in vitro infection, yielding around three amastigotes/cell (Figs. 2 and 3A, 3B). For the latter DTU, biological properties associated with low parasitemia, virulence, and pathogenicity have been identified. Additionally, it is hypothesized that its presence in human infections may result from the accidental introduction of vectors into the human food chain or contact between these vectors and humans due to environmental changes (Marcili et al. 2009; Monteiro et al. 2012).

Regarding metacyclic production, TcIII showed a count of 6,847,222 (MTs/mL) (Fig. 1C). Although life cycle information on the vector was successfully gathered for this DTU, infection experiments with Vero cells did not yield substantial quantities of CDTs. This outcome aligns with the rare occurrences of infection in humans by this DTU. TcIII is widely dispersed and occurs in small, focal transmission cycles, with distribution in Brazil and from western Venezuela to the Chaco region of Argentina (Barros et al. 2017). Up to date, only a few cases have been documented in Colombia, with armadillos identified as the primary reservoir (Altcheh et al 2019; Ramírez et al. 2010; Zingales 2018).

In the case of TcVI, it reached a peak production of 6,966,666 MTs/mL, coupled with a yield of 6 amastigotes/cell (Figs. 1D and 3A). These findings align with Oliveira’s 2017 study, where in vitro analyses of TcI, TcII, and TcVI demonstrated a lower performance for TcVI (Oliveira et al. 2017). The epidemiological significance of TcVI stands out as one of the most prevalent in human infections. This prevalence can be mainly attributed to the heightened risk of exposure and transmission to humans, as TcVI is primarily located near humans within domestic cycles. Highly distributed in southern and central regions of South American countries, TcVI has been associated with chronic CD, digestive manifestations, and vertical transmission, prompting speculation about a potential tropism in this DTU (Altcheh et al. 2019; Zingales 2018; Zingales et al. 2012).

The DTUs that demonstrated the highest potential for metacyclogenesis in this study were TcII (Y) and TcI (MG), with a mean of 24,194,444 MTs/mL and 52,000,489 MTs/mL, respectively (Fig. 1C, D). However, in the infection assessment, TcII ranked third, while both TcI strains lead the way, demonstrating rapid invasion and elevated amastigote production compared to the other DTUs (Figs. 2 and 3A, 3B). The performance of TcI and TcII has been previously assessed, yielding divergent results. Sales-Campos et al. identified higher blood parasitemias for TcII in a murine model, a finding supported by Peña et al., who observed a higher prevalence of TcII in the blood during mixed infections (Pena et al. 2011; Sales-Campos et al. 2015).

The variations in performance may be attributed to the in vitro nature of the experimental model. While both DTUs can infect Vero cells, the dynamics in the murine model differ significantly, with TcII exhibiting marked effectiveness. This difference could be related to a higher expression of heat shock proteins, cell surface molecules, and other molecules associated with stress resistance, as well as the distinct interactions between the parasite and the host immune system (Oliveira et al. 2017; Tavares de Oliveira et al. 2018). In contrast, the research conducted by Botero and colleagues in 2007 emphasized a higher prevalence of TcI strains in both blood and tissues, suggesting greater virulence associated with this DTU. Correspondingly, we found that the behavior of TcI in both cycles exhibited higher performance, positioning it with the most elevated metacyclogenesis potential and the highest CDT production (Figs. 1D and 3C) (Botero et al. 2007). Specifically, there were 792,500 CDT/mL for DA and 336,666 for MG. This phenomenon could be the root cause of the widespread distribution and prevalence of TcI in various domestic and sylvatic cycles. Moreover, it justifies its predominant role as the primary culprit for the vast majority of human infections in the Amazon basin and partially in the countries of the Southern Cone of South America (Abolis et al. 2011; Brenière et al. 2012).

From the 5th day of metacyclogenesis onward, statistically significant differences emerged between the two analyzed TcI strains, highlighting variations in peak MT levels and reaching their maximum on different days (Fig. 1D; Table S1). It is essential to note that the divergent behavior of the two TcI strains may be due to the extensive intra-DTU diversity. This diversity has prompted some researchers to propose a subdivision of this DTU based on observed differences in the intergenic region of the miniexon gene. This approach has revealed a broad spectrum of TcI genotypes, with specific genotypes notably associated with human infection (Guhl & Ramírez 2011).

The approach employed in this study has centered on a singular cell model, limiting the exploration of the diverse tropisms potentially manifested by the DTUs. This restriction may introduce discrepancies in the observed behavior within this specific context. Consequently, based on this research, it is necessary to assess the behavior of these DTUs across various cellular models. Moreover, it is crucial to delve into the performance of distinct strains in future investigations to establish a comprehensive understanding of the biological characteristics exhibited by these DTUs.

Nevertheless, it is crucial to emphasize that this study marks an initial exploration into the impact of genetic diversity. Essentially, the results not only shed light on the complexity of CD but also enhance our understanding of *Trypanosoma cruzi* biology and emphasize the phenotypic implications of its biological diversity. These findings highlight the critical importance of genotyping and advocate for studies that develop effective strategies for controlling and treating CD in diverse geographic regions.

## Conclusion

The variations in metacyclogenesis and infection dynamics not only occurred among different DTUs but also within strains, significantly impacting the biological cycles of *T. cruzi*. These differences could influence the parasite’s circulation, geographic distribution, and transmission. A deeper understanding of these processes provides crucial tools for comprehending ecoepidemiology and identifying key factors affecting the adaptation and spread of the parasite in various environments.

### Supplementary Information

Below is the link to the electronic supplementary material.

Supplementary file1 (PDF 155 KB)

## Data Availability

The data generated in this manuscript is within the main and supplementary files.
