# Supplementary material for: Comparative analysis of metacyclogenesis and infection curves in different discrete typing units of Trypanosoma cruzi
Source: Parasitol Res. 2024 Apr 11;123(4):181. doi: 10.1007/s00436-024-08183-4 (PMC11008065; doi:10.1007/s00436-024-08183-4)
Supplement: Supplementary file 1 — Supplementary file1 (PDF 155 KB) [file 436_2024_8183_MOESM1_ESM.pdf]

Table S1. *Tukey's multiple testing for metacyclogenesis in different DTUs.*

Tukey's multiple comparisons test	Significant	Adjusted P Value	Tukey's multiple comparisons test	Significant	Adjusted P Value
**Day 1**					
TcI (MG) vs. TcII (Y)	*	0.0197	**Day 9**		
TcI (MG) vs. TcIII	*	0.0254	TcII (Y) vs. TcIII	*	0.0141
**Day 3**			TcII (Y) vs. TcIV	**	0.0025
TcI (DA) vs. TcII (Y)	**	0.0018	TcII (Y) vs. TcVI	*	0.0284
TcI (DA) vs. TcVI	*	0.0189	TcIII vs. TcIV	**	0.0064
**Day 4**			TcIII vs. TcVI	*	0.0135
TcIII vs. TcIV	**	0.0066	TcIV vs. TcVI	**	0.005
TcIV vs. TcVI	*	0.0443	**Day 10**		
**Day 5**			TcI (DA) vs. TcI (MG)	*	0.0336
TcI (DA) vs. TcI (MG)	*	0.0184	TcI (DA) vs. TcIII	*	0.0303
TcI (DA) vs. TcII (Y)	**	0.0033	TcI (MG) vs. TcII (Y)	*	0.0408
TcI (MG) vs. TcII (Y)	**	0.0027	TcI (MG) vs. TcIII	*	0.0446
TcI (MG) vs. TcIII	**	0.0028	TcI (MG) vs. TcIV	*	0.0352
TcI (MG) vs. TcIV	**	0.0049	TcI (MG) vs. TcVI	*	0.041
TcI (MG) vs. TcVI	*	0.0161	TcIII vs. TcIV	**	0.0025
TcII (Y) vs. TcIII	**	0.0012	TcIV vs. TcVI	*	0.0128
TcII (Y) vs. TcIV	***	0.0009	**Day 11**		
TcII (Y) vs. TcVI	**	0.0042	TcI (DA) vs. TcI (MG)	*	0.0397
**Day 6**			TcI (MG) vs. TcII (Y)	*	0.0395
TcI (DA) vs. TcII (Y)	*	0.0197	TcI (MG) vs. TcIII	*	0.0389
TcII (Y) vs. TcIII	**	0.0055	TcI (MG) vs. TcIV	*	0.0394
TcII (Y) vs. TcIV	**	0.0038	TcI (MG) vs. TcVI	*	0.0447
TcII (Y) vs. TcVI	*	0.0156	TcIV vs. TcVI	*	0.0338
**Day 7**			**Day 12**		
TcI (DA) vs. TcII (Y)	*	0.0192	TcI (DA) vs. TcI (MG)	***	0.0009
TcII (Y) vs. TcIII	*	0.0331	TcI (DA) vs. TcIV	**	0.0049
TcII (Y) vs. TcIV	*	0.0215	TcI (MG) vs. TcII (Y)	***	0.0007
TcII (Y) vs. TcVI	*	0.0119	TcI (MG) vs. TcIII	***	0.0007
TcIII vs. TcIV	**	0.0053	TcI (MG) vs. TcIV	***	0.0007
**Day 9**			TcI (MG) vs. TcVI	***	0.0006
TcI (DA) vs. TcII (Y)	**	0.0022	TcII (Y) vs. TcIV	*	0.0446

Table S2. *(A) Multiple Tukey test for amastigogenesis (B) Production of cell-derived trypomastigotes at different DTUs*

A.

Tukey's multiple comparisons test	Significant	Adjusted P Value	Tukey's multiple comparisons test	Significant	Adjusted P Value
**Day 1**			**Day 4**		
TcI (MG) vs. TcIV	*	0.0186	TcI (DA) vs. TcVI	***	0.0004
TcII (Y) vs. TcIV	*	0.0301			
**Day 2**			TcI (MG) vs. TcII (Y)	**	0.0044
TcI (MG) vs. TcIV	****	<0,0001	TcI (MG) vs. TcIV	**	0.0042
TcI (MG) vs. TcVI	****	<0,0001	TcI (MG) vs. TcVI	***	0.0008
TcII (Y) vs. TcIV	****	<0,0001	TcII (Y) vs. TcIV	*	0.0103
TcII (Y) vs. TcVI	***	0.0001	TcII (Y) vs. TcVI	**	0.0071
**Day 3**			**Day 5**		
TcI (MG) vs. TcII (Y)	*	0.0182	TcI (DA) vs. TcI (MG)	**	0.0084
TcI (MG) vs. TcIV	**	0.0075	TcI (DA) vs. TcIV	*	0.0125
TcI (MG) vs. TcVI	**	0.0048	TcI (DA) vs. TcVI	**	0.0081
TcII (Y) vs. TcIV	**	0.0045	TcI (MG) vs. TcII (Y)	**	0.0084
TcII (Y) vs. TcVI	**	0.0031	TcI (MG) vs. TcIV	**	0.0075
**Day 4**			TcI (MG) vs. TcVI	**	0.0051
TcI (DA) vs. TcII (Y)	*	0.0121	TcII (Y) vs. TcIV	**	0.0064
TcI (DA) vs. TcIV	***	0.0009	TcII (Y) vs. TcVI	**	0.0024

B.

Tukey's multiple comparisons test	Significant	Adjusted P Value
**Day 4**		
TcI (MG) vs. TcIV	**	0.0094
TcI (MG) vs. TcVI	*	0.0144
**Day 5**		
TcI (DA) vs. TcII (Y)	*	0.041
TcI (DA) vs. TcIV	*	0.0475
TcI (DA) vs. TcVI	*	0.0456
TcII (Y) vs. TcVI	*	0.0314
